# Evidence of Oxytosis/Ferroptosis in Niemann–Pick Disease Type C

**DOI:** 10.3390/ijms26072915

**Published:** 2025-03-23

**Authors:** Kayla L. Sanchez, Jeanyoung Kim, Jacob B. White, Andrew Tolan, Naren P. Rajagopal, Douglas W. Anderson, Alexandra N. Shin, Samuel D. Shin, Antonio Currais, David Soriano-Castell, Pamela Maher, Salvador Soriano

**Affiliations:** 1 Department of Pathology and Human Anatomy, School of Medicine, Loma Linda University, Loma Linda, CA 92354, USA; klsanchez@students.llu.edu (K.L.S.); jeankim@students.llu.edu (J.K.); jwhite01@students.llu.edu (J.B.W.); 1narenr@gmail.com (N.P.R.); douander@med.umich.edu (D.W.A.); alexandra.n.shin@gmail.com (A.N.S.); shins2@ccf.org (S.D.S.); 2 The Salk Institute for Biological Studies, 10010 North Torrey Pines Road, La Jolla, CA 92037, USA; acurrais@salk.edu (A.C.); dsorianocastell@salk.edu (D.S.-C.)

**Keywords:** Niemann–Pick disease, ferroptosis, cerebellum, neurodegeneration, J147

## Abstract

Niemann–Pick Disease Type C (NPC) is a hereditary neurodegenerative disease characterized by selective cell vulnerability, particularly affecting cerebellar anterior Purkinje neurons. These neurons exhibit a distinctive pattern of degeneration due to the loss of NPC1 and/or NPC2 protein function, progressively extending towards posterior cerebellar regions. Our study aimed to explore the early factors influencing this selective vulnerability of anterior Purkinje neurons in NPC. Oxytosis/ferroptosis, a novel form of regulated cell death, has been implicated in neurodegenerative diseases, with its inhibition showing promising therapeutic potential. Our laboratory has previously identified parallels between NPC cellular pathology and ferroptotic markers, including elevated levels of lipid peroxidation and iron, mitochondrial dysfunction, and Ca^2+^ dyshomeostasis. However, whether oxytosis/ferroptosis underlies NPC cellular pathology remains unexplored. We hypothesize that loss of NPC1 function increases vulnerability to ferroptosis and that anti-ferroptotic compounds will reverse NPC cellular pathology. Through bioinformatic analyses of pre-symptomatic *Npc1^−/−^* Purkinje neurons and in vitro studies using primary dermal fibroblasts derived from NPC patients, we provide evidence suggesting that oxytosis/ferroptosis may play a pathogenic role in NPC. These findings highlight the potential of anti-ferroptotic compounds as a promising therapeutic strategy to mitigate neurodegeneration in NPC and potentially other related disorders.

## 1. Introduction

Niemann–Pick Disease Type C (NPC) is a rare neurodegenerative disorder caused by mutations in the genes *NPC1* (Chr.18q11.2) and/or *NPC2* (Chr.14q24.3), with *NPC1* mutations accounting for 95% of cases. While the precise roles of the NPC1 and NPC2 proteins are still under investigation, it is suggested that they serve as lipid transporters from the late endosome/lysosome to various cellular compartments [1].

The initial clinical presentation of NPC is widely heterogeneous; however, most cases result in progressive demyelination and neuronal atrophy [2]. Cell death occurs more prominently in the anterior cerebellar lobe where Purkinje neurons, the sole output of the GABAergic neuronal pathway, exhibit high vulnerability to the loss of NPC1 [3,4]. Consequently, individuals experience motor, neurological, and cognitive deficiencies, which diminish quality of life and eventually result in premature death [5].

The anterior-to-posterior patterns of cerebellar Purkinje cell death observed in NPC are not unique to this condition. Instead, similar gradients of vulnerability are evident in multiple cerebellar degenerative disorders, with lobule X exhibiting a natural resistance [6]. These pathological similarities suggest potential commonalities among these diseases. If a shared mechanism does underlie these patterns, elucidating the pathways involved in NPC cell death could also provide valuable insights into the molecular basis of other cerebellar diseases and shed light on the complex biology of Purkinje neurons.

The prevailing hypothesis that NPC neurodegeneration is primarily driven by impaired cholesterol transport has been a focal point for research; however, cholesterol-lowering therapies have not yielded significant benefits in slowing neurodegeneration [4]. Additionally, mouse studies have shown that while cholesterol levels are uniformly elevated across all *Npc1^−/−^* cerebellar lobes, cell viability does not follow this pattern [7], indicating that other factors likely play a critical role in neurodegeneration.

Recent evidence points to a complex cellular pathology in NPC, characterized by lipid peroxidation, oxidative stress, elevated iron, and Ca^2+^ dyshomeostasis [8,9,10,11]. These conditions are reminiscent of a novel non-apoptotic regulated cell death pathway known as oxytosis/ferroptosis. Initially termed oxytosis [12], the process was renamed as ferroptosis to incorporate its iron-dependent nature [7]. Oxytosis/ferroptosis is traditionally characterized by a disruption in the cystine/glutamate transporter (system X_c_^-^) or the glutathione (GSH)/ glutathione peroxidase 4 (GPx4) pathway. This leads to the accumulation of toxic lipid peroxides [9] due to the inability of GPx4, often due to a loss of its essential substrate GSH, to convert these peroxides into safe lipid alcohols [13]. Additionally, the Fenton reaction, where excessive ferrous iron (Fe^2+^) and reactive oxygen species (ROS) interact, can initiate oxytosis/ferroptosis by generating highly reactive hydroxyl radicals that can oxidize lipids [14].

Despite these mechanisms being well documented in other contexts, an analysis of their role in NPC brain pathology is lacking. Here, we propose that oxytosis/ferroptosis may be an early critical contributor to neurodegeneration in NPC.

**Figure 1:**
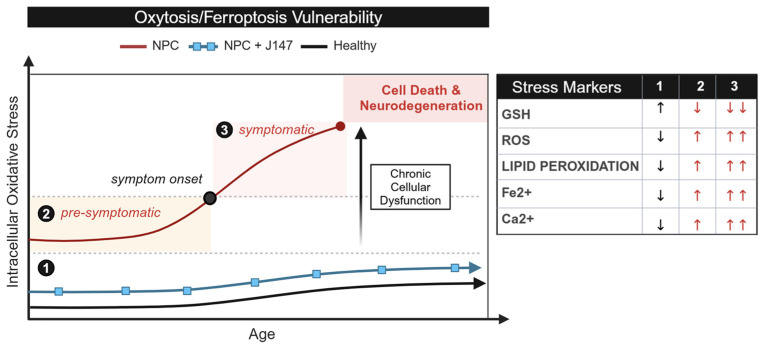
Proposed vulnerability of Niemann–Pick and healthy cells to oxytosis/ferroptosis. In NPC cells, a compromised antioxidant defense system (i.e., GSH reduction) leads to elevated baseline levels of lipid peroxides and reactive oxygen species (ROS). This imbalance predisposes NPC cells to oxytosis/ferroptosis more readily than healthy cells when exposed to intrinsic stress over time. In this scenario, the addition of anti-oxytotic/ferroptotic compounds, such as J147, has the potential to reduce the baseline level of oxytotic/ferroptotic stress close to that present in healthy, age-matched cells. ↑ indicates increased levels. ↓ indicates decreased levels. ↑↑ and ↓↓ indicate significantly increased or significantly decreased levels, respectfully.

A study by Ko et al. (2005) revealed a notable disparity in cell death among Purkinje neurons in cerebellar lobules I–III versus lobule VI of *Npc1^−/−^* mice [7]. Building on this observation, we propose the concept of a chronic, sublethal level of oxytosis/ferroptosis to explain the differential vulnerability to neurodegeneration in the NPC cerebellum. As illustrated in Figure 1, we hypothesize that loss of NPC1 function leads to reduced antioxidant defenses (i.e., reduced GSH levels) and a subsequent increase in intracellular oxidative stress in Purkinje neurons. This imbalance begins before symptom onset and accumulates over time, causing chronic cellular dysfunction that remains at sublethal levels. However, as patients age, the oxidative stress imbalance reaches a critical, irreversible point, leading to ferroptotic cell death. If that scenario is accurate, and oxytosis/ferroptosis is an early pathogenic factor in NPC, it would logically follow that Purkinje neurons in anterior lobules (I–III) are closer to this lethal threshold than those in more posterior lobules, consistent with the observed pattern of neurodegeneration.

As a corollary, confirmation of oxytosis/ferroptosis activation in the more vulnerable anterior lobules would conceivably open the possibility for anti-oxytotic/ferroptotic therapeutic strategies to delay pathogenesis by mitigating sublethal levels of oxytotic/ferroptotic stress to those closer to the levels in the posterior lobes. In that regard, the compound J147, generated in the laboratories of Dr. David Schubert and Pamela Maher, is effective in reversing oxytotic/ferroptotic pathology in animal models of Alzheimer’s Disease [13,14]. J147 is an inhibitor of α-F1-ATP Synthase 5 (ATP5a) which reduces free fatty acids [15] and oxidative stress, maintains mitochondrial homeostasis [16], and stabilizes Ca^2+^ flux against glutamate and Ras-Selective Lethal 3 (RSL3), an inducer of oxytosis/ferroptosis that inhibits GPx4 [17]. Because those cellular pathological hallmarks are also present in NPC, compounds such as J147 have the potential for therapeutic value in NPC.

In this study, we carried out in silico analysis of pre-symptomatic Purkinje neurons isolated from anterior and posterior regions of the *Npc1^−/−^* cerebellum and provide evidence for higher vulnerability to oxytosis/ferroptosis in lobules I–III when compared to lobules VI and X, consistent with our sublethal threshold model of neurodegeneration in NPC (Figure 1). In addition, using dermal fibroblasts derived from NPC patients, we provide mechanistic evidence showing that NPC cells exhibit a constitutively higher pro-oxytotic/ferroptotic environment than age-matched healthy controls, also consistent with our model.

## 2. Results

### 2.1. Oxytosis/Ferroptosis Is Upregulated in NPC Purkinje Cells of Cerebellar Lobule III

Clinically, the NPC anterior cerebellum is the first region to exhibit cellular injury [15]. We hypothesize that this selective phenomenon reflects a differential vulnerability to oxytosis/ferroptosis in Purkinje neurons across individual cerebellar lobules.

To investigate this further, we retrieved a subset of transcriptomic data isolated from cerebellar lobules III, VI, and X of *Npc1^−/−^* (BALB/cNctr-Npc1miN/J) and control mice generated by Martin et al. (2020) [17]. In NPC, as observed in other cerebellar diseases, the anterior lobules (I–III) exhibit greater vulnerability, whereas lobule X has a higher degree of resistance [16]. We performed a secondary analysis of these *Npc1^−/−^* cerebellar lobule datasets [17] to evaluate the presence of differential oxytotic/ferroptotic pathway activation that might correlate with neurodegeneration vulnerability.

**Figure 2:**
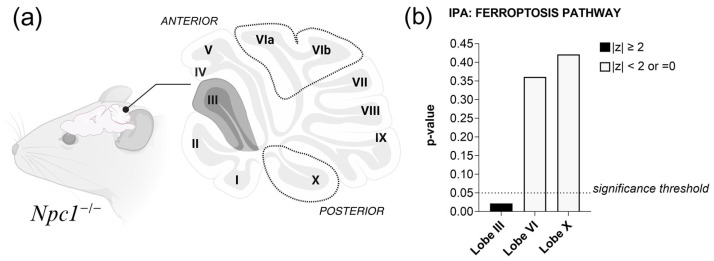
IPA Core Analysis of pre-symptomatic *Npc1^−/−^* cerebellar lobules. (**a**) Anatomical illustration of the *Npc1^−/−^* mouse cerebellum, depicting regions of interest potentially affected by oxytosis/ferroptosis. (**b**) Results of the IPA Ferroptosis Pathway show significant upregulation in lobule III of the pre-symptomatic *Npc1^−/−^* cerebellum (z-score= 2.00; *p*-value 2.23 × 10^−2^) when compared to the control cerebellum lobule III. *Npc1^−/−^* cerebellar lobules VI (z-score= 0; *p*-value 3.61 × 10^−1^) and X (z-score= 0; *p*-value 4.21 × 10^−1^) are not significant compared to their control counterparts. Significance is determined by a z-score of ≥ 2.00 and *p*-value < 0.05.

Using Ingenuity Pathway Analysis (IPA), we discovered that the *Npc1^−/−^* cerebellar lobule III contains significant upregulation of oxytosis/ferroptosis genes (*p*-value < 0.05, z-score = 2.00) when compared to wildtype cerebellar lobule III (Figure 2b). In contrast, *Npc1^−/−^* cerebellar lobules VI (z-score = 0; *p*-value > 0.05) and X (z-score = 0; *p*-value > 0.05) showed no significant differences within the oxytosis/ferroptosis pathway when compared to their respective healthy controls (Figure 2b). These findings are consistent with our working model proposing the presence of sublethal levels of pro-oxytotic/ferroptotic stress (Figure 1) in the most vulnerable lobules of the NPC cerebellum. It is also noteworthy that the mice evaluated in this study were pre-symptomatic, suggesting that oxytosis/ferroptosis may be an early, and therefore therapeutically valuable, mechanism contributing to neurodegeneration in the NPC brain.

**Figure 3:**
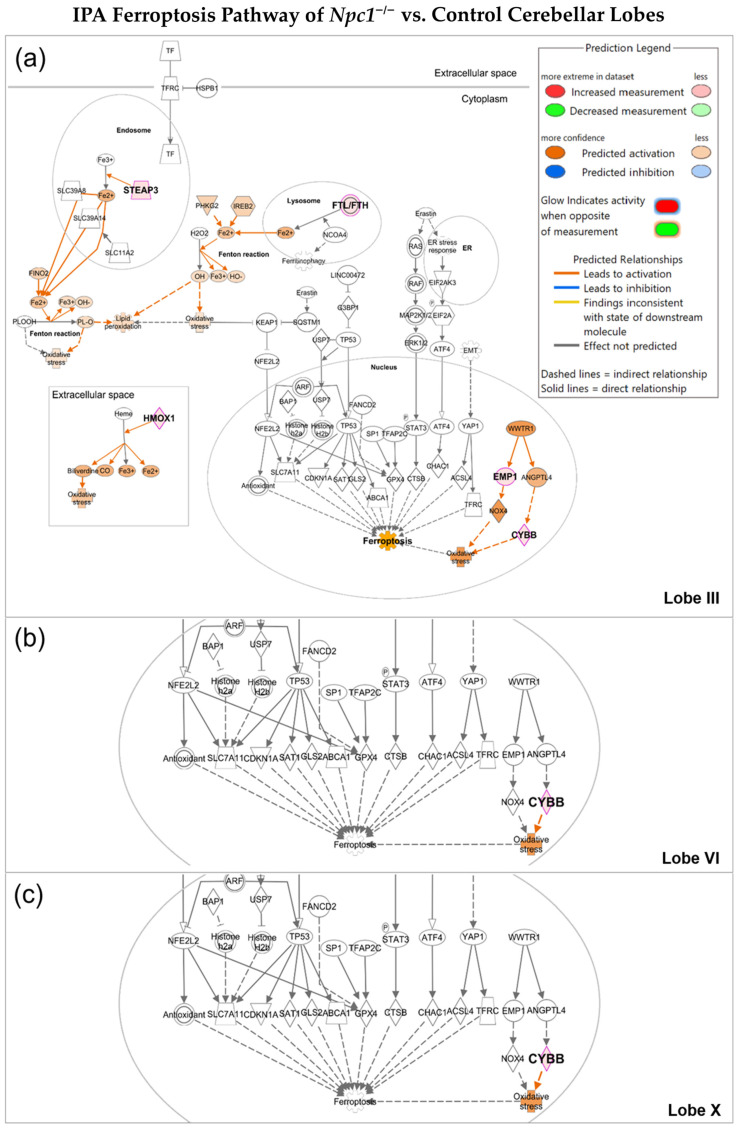
Overview of IPA Oxytosis/Ferroptosis Pathway. (**a**) *Npc1^−/−^* lobule III shows significant upregulation of genes reflecting iron dyshomeostasis and elevated oxidative stress when compared to wildtype control. Cybb (Nox2) is upregulated in lobules VI (**b**) and X (**c**), and both show no evidence of iron imbalance or upregulation of Nox4.

To gain a more detailed understanding of the genes involved in initiating oxytosis/ferroptosis, we further investigated the oxytosis/ferroptosis pathway within NPC cerebellar lobules III, VI, and X. (Figure 3). Of the genes significantly upregulated in cerebellar lobule III, four areas were implicated in oxytosis/ferroptosis activation: the extracellular space, lysosome, endosome, and nucleus (Figure 3a). Of the 136 genes associated with the oxytosis/ferroptosis pathway, *Npc1^−/−^* cerebellar lobule III had 5 significantly upregulated genes: Ftl, Steap3, Hmox1, Cybb (Nox2), and Emp1. Three of these differentially expressed genes are significantly linked to iron regulation. Steap3 metalloreductase (Steap3) and heme oxygenase 1 (Hmox1) can contribute to the production of intracellular ferrous ions (Fe^2+^), and increases in ferritin light chain levels (Ftl) can be a reflection of increases in intracellular iron. The increased iron can contribute to the production of ROS and lipid peroxides, as depicted in Figure 3a. Two DEGs additionally contribute to the production of ROS. Cybb, cytochrome b-245 (Nox2), is a subunit of the NADPH oxidase complex and serves as a producer of superoxide [18,19]. Emp1 regulates the expression of Nox4 that generates H_2_O_2_. By contrast, both lobules VI and X exhibit upregulation of a single gene, Cybb (Figure 3b,c). While Cybb may be responsible for ROS generation, this alone may not be sufficient to induce oxytosis/ferroptosis. Instead, ROS levels may remain below the sublethal threshold described in our working model (Figure 1).

Additional analysis highlighting the differences between lobule III and lobules VI and X is presented in Supplemental Figures S2–S4. In contrast to *Npc1^−/−^* cerebellar lobule III, which exhibits dysregulated iron homeostasis (Supplemental Figure S2), lobules VI and X do not show evidence of altered iron handling or other cellular pathways associated with oxytosis/ferroptosis in these lobules (Supplemental Figures S3 and S4).

### 2.2. NPC Cells Are More Vulnerable to Ferroptosis than Age-Matched Controls

Despite their physiological differences, dermal fibroblasts have been widely recognized as a valuable model for studying cellular mechanisms relevant to brain cells, including lipid peroxidation, Ca^2+^ dyshomeostasis, mitochondrial dysfunction, and immune responses [8,9,10,11]. Leveraging this established model, we utilized dermal fibroblasts from NPC patients and age-matched controls to test our hypothesis that a heightened baseline of oxytotic/ferroptotic stress in NPC cells contributes to increased susceptibility to oxytotic/ferroptotic death as patients age (Figure 1).

**Figure 4:**
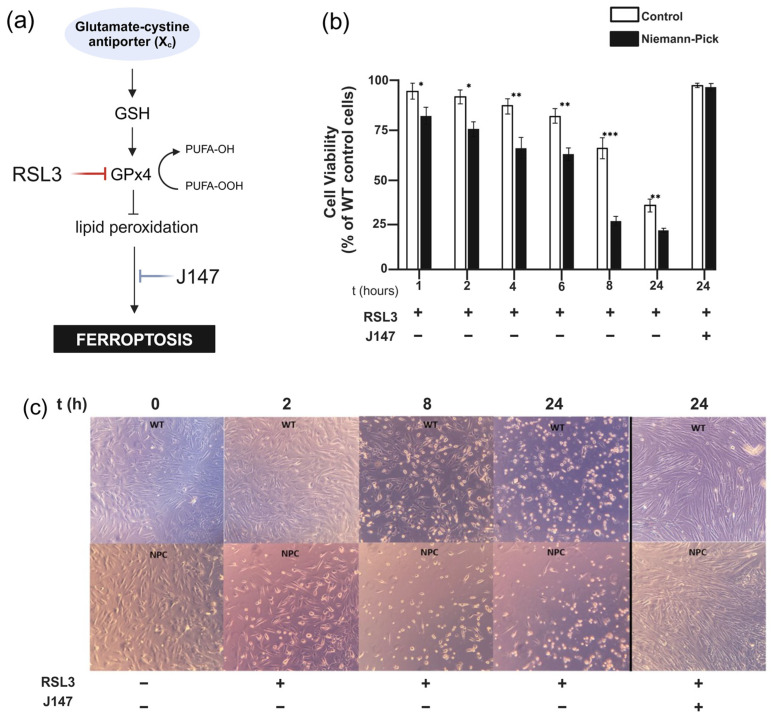
NPC cells show increased vulnerability to ferroptosis. (**a**) Overview of drug targets used in this study and their effects on the oxytosis/ferroptosis pathway. (**b**) MTT assay showing NPC fibroblasts died at a higher rate at all timepoints, with the most significant difference at 8 h. Error bars display SEM. Student’s *t*-test was used to calculate statistical differences between NPC 6-month and WT 1-year fibroblasts (* *p* < 0.05; ** *p* < 0.01; *** *p* < 0.001). Data are from 3 independent experiments performed in triplicate. (**c**) Representative image of NPC and healthy cells treated with 100 nM RSL3 and/or 1 μM J147.

In the context of our model, we reasoned that additional exposure to oxytotic/ferroptotic stress would reveal a higher number of NPC cells dying by oxytosis/ferroptosis than age-matched control cells, because of the higher baseline of oxytotic/ferroptotic stress in NPC (Figure 1). To begin to test this hypothesis, we treated dermal fibroblasts from NPC patients and controls with Ras-selective Lethal 3 (RSL3), a ferroptosis inducer that inhibits GPx4 activity, thereby impairing antioxidant defenses against lipid peroxidation [20,21] (Figure 4a). If NPC cells are indeed closer to an irreversible threshold of oxytotic/ferroptotic death (Figure 1), the additional oxytotic/ferroptotic stress generated by RSL3 treatment would be expected to push a greater number of NPC cells beyond that threshold. To align with our previous studies investigating the role of RSL3 in oxytosis/ferroptosis in cultured cells [22,23], we treated cells with 100 nM RSL3 at various timepoints up to 24 h and quantified cell death using the MTT assay. Figure 4b shows the effect of RSL3 on cell survival in NPC cells (6 months old; #GM17923) and healthy controls (1 year old; #GM05659). Differences in vulnerability to RSL3 are measurable at all timepoints. Figure 4c provides a visualization of the morphological changes in cells exposed to RSL3 at the corresponding time. Notably, co-treatment with the anti-oxytotic/ferroptotic compound J147 completely reversed cell death induced by RSL3 (Figure 4b,c), confirming that oxytosis/ferroptosis is the primary mechanism of cell death in this experimental context. Supplemental Figure S1 provides a comparable analysis of NPC and control cells from different age-matched individuals, further highlighting the differential response to RSL3, with NPC cells displaying increased vulnerability.

These results collectively demonstrate that NPC cells exhibit heightened vulnerability to oxytosis/ferroptosis compared to healthy controls, aligning with our working model that posits an elevated baseline of intracellular oxidative stress and, consequently, oxytotic/ferroptotic stress in NPC cells (Figure 1).

### 2.3. Glutathione Peroxidase 4 (GPx4) Is Lower in NPC Compared to Age-Matched Controls

**Figure 5:**
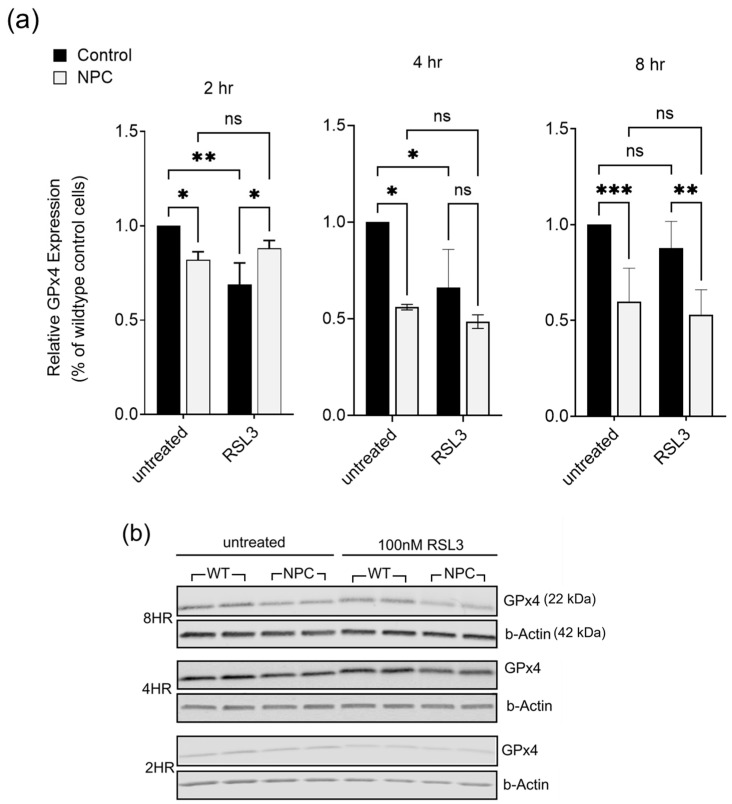
NPC cells express lower Glutathione Peroxidase 4 (GPx4) levels. (**a**) Relative levels of GPx4 expression across 2, 4, and 8 h following 100 nM RSL3 treatment. Experiments were run in duplicates (n = 3 per timepoint). Data were normalized to β-actin and two-way ANOVA was performed (* *p* < 0.05; ** *p* < 0.01; *** *p* < 0.001). ns: non-significant (**b**) Representative images of Western blots for 6-month-old NPC (#GM17923) and 1-year-old wildtype control (#GM05659).

GPxs are a group of antioxidant enzymes that protect against oxidative stress, namely the reduction of free hydrogen peroxide to water and lipid peroxides into alcohol [22]. Among the eight isoforms that exist, GPx4 is the only enzyme capable of reducing esterified oxidized fatty acids and cholesterol hydroperoxides into non-toxic lipid alcohols, which has established it as a key player in the oxytosis/ferroptosis pathway [24]. To assess GPx4 expression as a possible contributor to oxytosis/ferroptosis sensitivity in NPC, Western blot analyses were conducted using dermal fibroblasts from NPC patients and controls following treatment with 100 nM RSL3 at 2, 4, and 8 h timepoints. Timepoints for 12 and 24 h were excluded from this analysis due to the high levels of cell death. In untreated (vehicle only) NPC cells, there was consistently lower expression of GPx4 compared to controls (Figure 5). Wildtype control cells exhibited a decrease in GPx4 levels at 2 and 4 h following treatment with RSL3, consistent with previously reported responses to RSL3-induced ferroptosis [25]. By 8 h, GPx4 levels in wildtype cells increased, potentially reflecting the activation of a protective mechanism to counteract ferroptotic stress. In contrast, NPC cells failed to show any increase in GPx4 levels after RSL3 treatment. This inability to upregulate GPx4 suggests that NPC cells, possibly due to heightened sublethal oxytotic/ferroptotic stress, are unable to activate an effective protective response.

## 3. Discussion

Effective treatments for NPC remain a significant challenge. While cholesterol buildup has long been proposed as the primary pathogenic trigger, therapies targeting cholesterol have largely failed to improve neurological symptoms, despite reducing systemic cholesterol levels [25,26].

Among the therapies under investigation, 2-hydroxypropyl-β-cyclodextrin (HP-β-CD), a widely used carrier molecule for lipophilic drugs, has shown limited success in clinical trials, with insufficient data on its long-term efficacy [25,26]. Recent advances include the FDA approval of Arimoclomol [27], a heat shock response activator, for use in combination with Miglustat, a glycolipid synthesis inhibitor. While Miglustat alone has only shown modest improvements in NPC [28], this combination therapy restores lysosomal function [29]. However, this approach has only been tested in patients with mild neurological symptoms [28].

In September 2024, N-acetyl-L-leucine (NALL) received approval for use in the United States. NALL offers a novel therapeutic approach by enhancing lysosomal and mitochondrial function, modulating ATP production, restoring neuronal membrane potential, and reducing neuroinflammation [30,31].

While these recent therapeutic developments mark a promising step forward in NPC symptom management [29,30], they remain palliative, addressing consequences rather than root causes of the disease, and understanding the fundamental molecular and cellular mechanisms driving NPC neurodegeneration remains a key objective for the development of evidence-based therapies. Here, we propose the hypothesis that NPC pathology may be driven, at least in part, by the early activation of the regulated cell death pathway known as oxytosis/ferroptosis. Our hypothesis is based on the shared characteristics of NPC and oxytosis/ferroptosis, including increased lipid peroxidation, elevated levels of iron, mitochondrial dysfunction, and Ca^2+^ dyshomeostasis [8,9,10,11]. Furthermore, we hypothesize that the activation of cell death by oxytosis/ferroptosis in NPC is not a binary event, but rather the culmination of iron-driven oxidative stress that contributes to the differential cell vulnerability as described in our sublethal threshold model (Figure 1). At early stages prior to reaching that threshold, this heightened vulnerability is potentially reversible, as our own data (Figure 4) and multiple studies in other neurodegenerative conditions [32,33] using the anti-oxytotic/ferroptotic compound J147 suggest. However, as oxidative stress continues to increase with age and the antioxidant systems become unable to counteract it, there is a transition to cell death (Figure 1).

Given the distinct spatiotemporal neurodegeneration in the cerebellum in NPC, which begins in the anterior lobules I–III and progresses to more posterior lobules, it is plausible that this pattern reflects varying levels of sublethal oxytotic/ferroptotic stress, as hypothesized in Figure 1. Higher oxytotic/ferroptotic stress in the anterior lobules, such as increased iron, lipid peroxidation, and ROS, may lead to earlier activation of cell death. Our secondary in silico analysis of pre-symptomatic *Npc1^−/−^* cerebellar data generated by Martin et al. (2020) [17] supports this model. Specifically, IPA analysis of *Npc1^−/−^* Purkinje neurons of anterior lobule III reveals significant activation of oxytosis/ferroptosis, whereas lobules VI and X are not affected (Figure 3). Among the genes upregulated in *Npc1^−/−^* lobule III, Steap3, Hmox1, and Flt are key regulators of iron homeostasis, while Emp1 and Cybb are involved in ROS generation. The dysregulation of iron homeostasis coupled with increased ROS production could thereby promote conditions favorable to an increase in lipid peroxidation [14]. In contrast to *Npc1^−/−^* anterior lobule III, cerebellar lobules VI (Figure 3b) and X (Figure 3c) both share a single upregulated gene in the oxytosis/ferroptosis pathway, Cybb, that alone is not predicted to increase oxytotic/ferroptotic cell death. Aligning with our hypothesis, we conclude that upregulation of genes involved in Fe^2+^ regualtion and ROS production supports the idea that the pre-symptomatic anterior lobule III is in an early state of sublethal oxytotic/ferroptotic stress while posterior lobules VI and X have yet to be affected in pre-symptomatic *Npc1^−/−^* mice.

Additional evidence of a link between oxytosis/ferroptosis and NPC was identified in hepatocytes [34] and HEI-OC1 cells [35]. While these findings were not reported in the brain, they represent additional evidence that NPC mutations can lead to ferroptosis. In addition, IP-10/CXCL10 impairs neuronal function in a ferroptosis-dependent manner and induces lipid peroxidation in neurons; these phenotypes are exacerbated by ferroptotic agents and reversed by ferroptosis inhibitors [36]. These findings are relevant because we have reported IP-10/CXCL10 in the *Npc1^−/−^* cerebellum as the only upregulated cytokine at 3 weeks of age, prior to symptom onset, therefore providing additional evidence for the role of ferroptosis early in NPC pathogenesis [9].

In summary, we demonstrate that NPC cells exist in a heightened oxytotic/ferroptotic environment, making them more vulnerable to cell death via this pathway. We propose that this vulnerability may help explain the selective neurodegeneration observed in anterior Purkinje neurons. Future research should prioritize validating these results in vivo and investigating the efficacy of anti-ferroptotic therapies, which could open new avenues for treating NPC and other neurodegenerative disorders associated with oxytosis/ferroptosis.

## 4. Materials and Methods

### 4.1. Cell Lines

Patient-derived NPC dermal fibroblasts (6 months old, #GM17923; 4 years old, #GM17921) and wildtype healthy controls (1 year old, #GM05659; 5 years old, #GM05381) were purchased from Coriell Institute for Medical Research (Camden, NJ, USA). Cell lines were cultured in Dulbecco’s Modified Eagle Medium (11965092;Thermo Fisher Scientific, Pittsburgh, PA, USA) supplemented with 5% fetal bovine serum (R&D Systems, Minneapolis, MN, USA) and 1% PenStrep (15140-122; Thermo Fisher Scientific). Cells were maintained in a humidified chamber at 37 °C with 5% CO_2_. For subsequent experiments, cell populations were seeded at ~30% confluency and treated at ~80% confluency.

### 4.2. Ingenuity Pathway Analysis

Transcriptomic data for cerebellar Purkinje neurons in lobules III, VI, and X of *Npc1^−/−^* (BALB/c*^Nctr-Npc1miN/J^*) and *Npc1^+/+^* mice were sourced from Martin et al. (2020) [17]. Differentially expressed genes (DEGs) from the wt-mut_III-III, wt-mut_VI-VI, and wt-mut_X-X datasets were selected based on an adjusted *p*-value < 0.05 and log2 fold change greater than 0.58 (>1.5-fold increase) or less than −0.58 (< 1.5-fold change), as described by Martin et al. (2020) [17]. DEGs from each dataset were imported into Ingenuity Pathway Analysis Version 24.0.2 (IPA, Qiagen, Redwood City, CA, USA) to look at Canonical Pathways and Mechanistic Maps. Significant activation or inhibition of a Canonical Pathway was determined by a z-score of |Z| ≥ 2 and *p*-value < 0.05.

### 4.3. MTT Cytotoxicity Assay

Cell viability was determined by the MTT Assay Kit (ab211091; Abcam, Cambridge, UK) according to the manufacturer’s instructions. NPC dermal fibroblasts and controls were harvested at approximately 80% confluency and seeded onto 96-well plates. Plates were incubated in a humidified chamber at 37 °C with 5% CO_2_ for 24 h. After incubation, the cell culture medium was discarded and treated with various doses of RSL3 (0.1, 0.25, 0.5 µM, or 1 µM) or vehicle alone. Following treatment for the indicated times, the cells were incubated with MTT at 37 °C for 3 h and then MTT solvent for 15 min. Absorbance was measured with a Spectra Max i3X and read with Soft Max Pro (version 6.5.1) at 590 nm. Percent cytotoxicity was calculated using (100 × (control-sample)/control).

### 4.4. Western Blot Analysis

Cells were lysed using 1x RIPA buffer (50 mM Tris-HCl, pH 8.0, 0.1% Triton X-100, 1% Sodium Deoxycholate, 0.1% SDS) and supplemented with Phosphatase Single-Use Inhibitor Cocktail 1: 10 (RC232651; Thermo Fisher Scientific, Pittsburgh, PA, USA). Cells were collected and sonicated 1× for 3 s. Samples were centrifuged at 13,000× *g* for 20 min at 4 °C. The supernatant was collected, and protein concentrations were determined using the Pierce BCA protein assay kit, following the manufacturer’s instructions. Cell protein lysates were separated on a 4–20% Novex Tris-Glycine Plus WedgeWell Gel using Invitrogen MiniBlot for 1 h at 120V. Proteins were transferred to a 0.45 μM nitrocellulose membrane (1212602; GVS North America, Sanford, ME, USA) for 45 min at 120V. Membranes were blocked with 3% milk in Tris-buffered saline with 0.05% Tween^®^ 20 Detergent (TBS-T) at room temperature for 1 h. Following 0.5% TBS-T washes (3×), membranes were probed overnight at 4 °C with GPx4 at 1:1000 (Abcam, 125066) with β-actin (MA1-140; Invitrogen, Waltham, MA, USA). The molecular weight of the protein bands was determined using a Color-coded prestained protein marker (74124S; Cell Signaling Technology, Danvers, MA, USA).

### 4.5. Statistical Analysis

Statistical analysis was performed using GraphPad Prism Version 10.2.0 (Graphpad Software, La Jolla, CA, USA). Data are represented as the mean ± SEM. Analyses were performed using an unpaired, two-tailed Student *t*-test in two-group comparisons or two-way ANOVA for multiple-group comparisons. Values of *p* < 0.05 were considered statistically significant (* *p* < 0.05; ** *p* < 0.01; *** *p* < 0.001).

## Data Availability

The raw data supporting the conclusions of this article will be made available by the authors on request.

## Supplementary Materials

The following supporting information can be downloaded at https://www.mdpi.com/article/10.3390/ijms26072915/s1.

## Abbreviations

The following abbreviations are used in this manuscript:

NPC	Niemann–Pick Disease Type C1
AD	Alzheimer’s Disease
