# Supplementary material for: Evidence of Oxytosis/Ferroptosis in Niemann–Pick Disease Type C
Source: Int J Mol Sci. 2025 Mar 23;26(7):2915. doi: 10.3390/ijms26072915 (PMC11988824; doi:10.3390/ijms26072915)
Supplement: Supplementary file 1 [file ijms-26-02915-s001.zip › ijms-3522846-supplementary.pdf]

Supplementary Materials

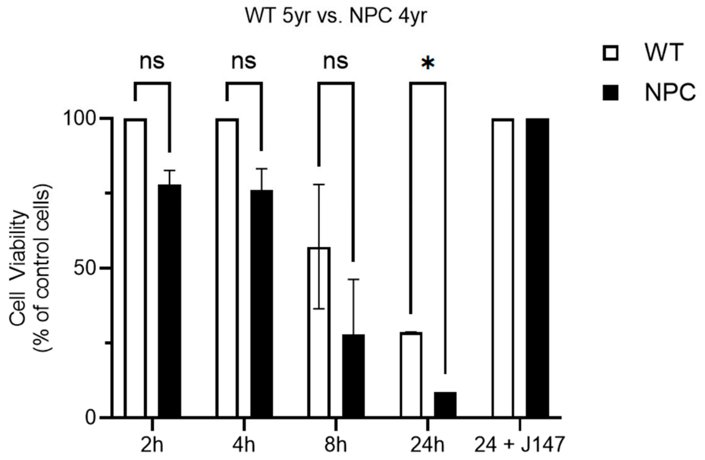

Figure S1. MTT Assay of NPC Human Dermal Fibroblasts and Healthy Controls

Effect of 100 nM RSL3 on the cell viability of primary dermal fibroblasts from an NPC patient (4 years old; #GM17921) and a healthy control (5 years old; #GM05381). Control fibroblasts showed no reduction in viability at 2 hours or 4 hours following RSL3 treatment, with a significant decrease observed only at 8 hours and 24 hours. In contrast, NPC fibroblasts exhibited a noticeable decline in viability as early as 2 hours and 4 hours, with a dramatic reduction at 8 hours and 24 hours. Although statistical significance is only reached at the 24-hour time point, clear trends at all earlier time points strongly support the notion, also illustrated in Figure 4, that NPC cells are more vulnerable to RSL3-induced stress compared to controls. Notably, co-incubation with the anti-ferroptotic compound J147 (1 μM) fully reversed toxicity in all cells, confirming that cell death occurs via oxytosis/ferroptosis. Error bars represent SEM. Statistical differences were calculated using Student's t-test (* $p < 0.05$).

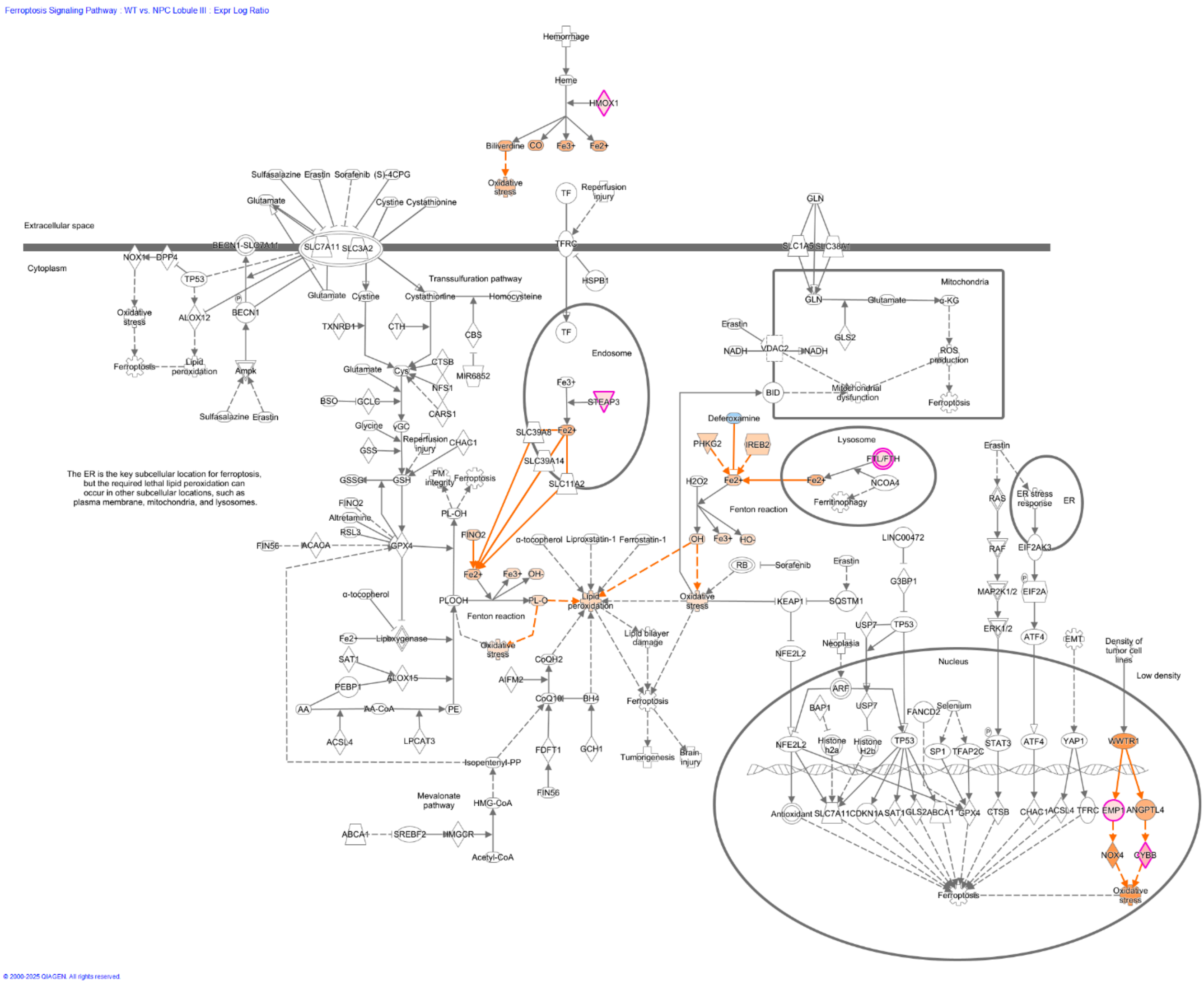

Figure S2. Overview of the IPA Ferroptosis Pathway in *Npc1*$^{-/-}$ Lobule III vs. Wildtype Control

Changes that drive the ferroptosis pathway are associated with upregulation of genes (light red) involved in iron-handling such as HmoxI, Steap3, and Ftl. Oxidative stress is generated through the Wwtr1-Emp1-Nox4 and Wwtr1-Angptl-Cybb upregulation.

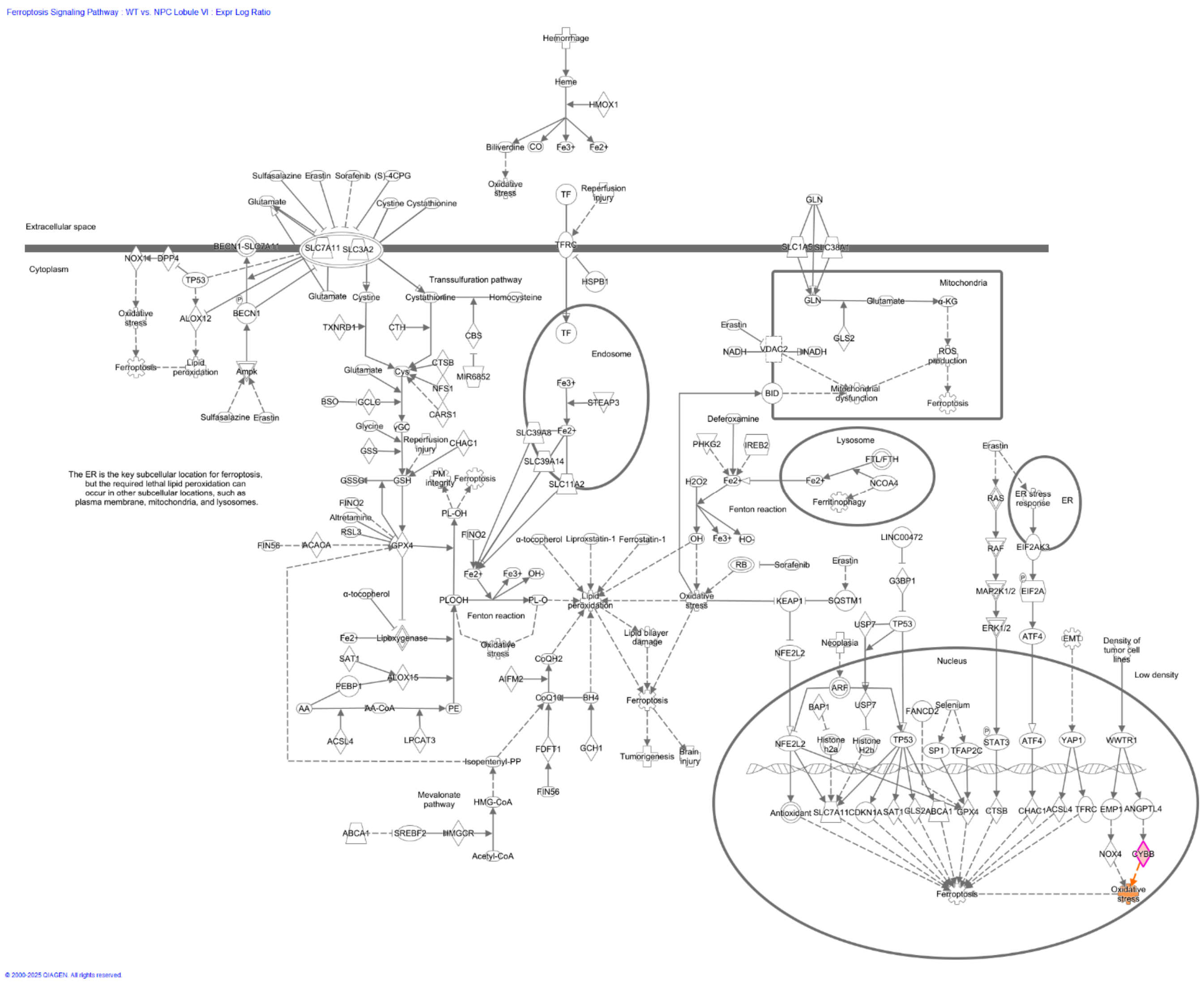

© 2000-2025 QIAGEN. All rights reserved

Figure S3. Overview of the Ferroptosis IPA Pathway in *Npc1*$^{-/-}$ Lobule VI vs. Wildtype Control

No significant changes were observed in other cellular pathways within *Npc1*$^{-/-}$ cerebellar lobule VI that could contribute to oxytosis/ferroptosis. The upregulation of a single gene, Cybb, was insufficient to activate the pathway.

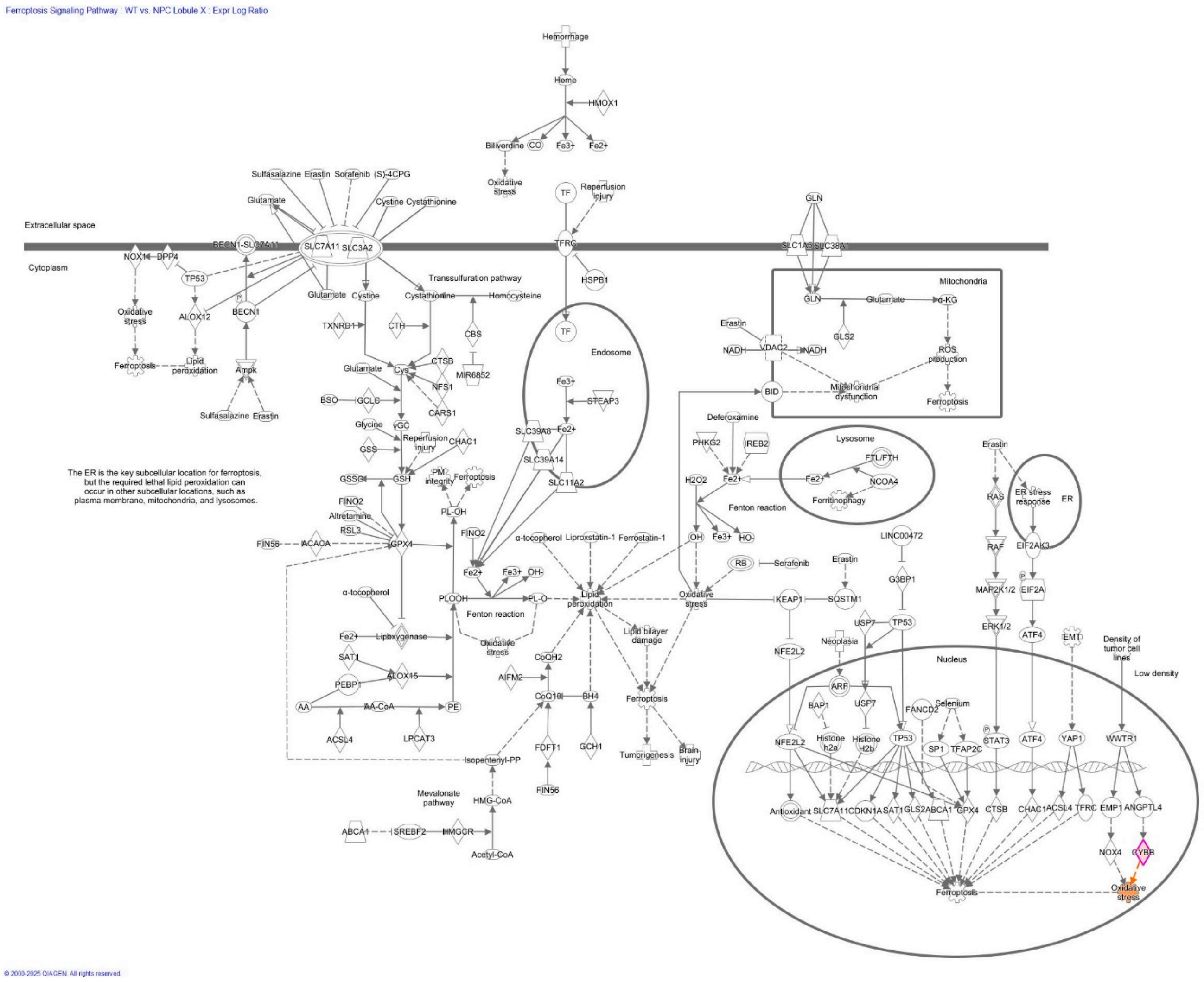

Figure S4. Overview of the IPA Pathway in *Npc1*$^{-/-}$ Lobule X vs. Wildtype Control

No significant changes were observed in other cellular pathways within *Npc1*$^{-/-}$ cerebellar lobule X that could contribute to oxytosis/ferroptosis. The upregulation of a single gene, Cybb, was insufficient to activate the pathway.
